# Combining canine mesenchymal stromal cells and hyaluronic acid for cartilage repair

**DOI:** 10.1590/1678-4685-GMB-2019-0275

**Published:** 2020-03-02

**Authors:** Maria Inês Wits, Gabriela Cabanas Tobin, Maiele Dornelles Silveira, Karine Gehlen Baja, Luisa Maria Macedo Braga, Patricia Sesterheim, Melissa Camassola, Nance Beyer Nardi

**Affiliations:** ^1^ Universidade Luterana do Brasil, Hospital Veterinário, Canoas, RS, Brazil.; ^2^ Universidade Luterana do Brasil, Laboratório de Células-Tronco e Engenharia de Tecidos, Canoas, RS, Brazil.; ^3^ CellMed Medicina Regenerativa, Porto Alegre, RS, Brazil.; ^4^ Fundação Universitária de Cardiologia, Instituto de Cardiologia do Rio Grande do Sul, Porto Alegre, RS, Brazil.

**Keywords:** Mesenchymal stromal cells, hyaluronic acid, osteoarthritis, dog

## Abstract

Cell therapy and tissue engineering have been intensively researched for repair of articular cartilage. In this study, we investigated the chondrogenic potential of canine adipose-derived mesenchymal stromal cells (ASCs) combined to high molecular weight hyaluronic acid (HA) *in vitro*, and their therapeutic effect in dogs with chronic osteoarthritis (OA) associated with bilateral hip dysplasia. Canine ASCs were characterized after conventional 2D culture or 3D culture in HA, showing adequate immunophenotype, proliferation and trilineage differentiation, as well as chondrogenesis after cultivation in HA. ASC/HA constructs were used to treat 12 dogs with OA, sequentially assigned to control, ASC and ASC/HA groups. Animals were examined for clinical, orthopedic and radiological parameters. Lameness at walk and pain on manipulation were reduced in the ASC group and mainly in the ASC/HA group. Range of motion and detection of crepitus on hip rotation and abduction improved similarly in all groups. For articular edema, muscle atrophy, Norberg angle values and radiographic analyses, there were no variations throughout the period. These results indicate that ASC/HA constructs are safe and may be an effective therapeutic tool in treating canine chronic osteoarthritis, which should be confirmed with larger studies and additional clinical parameters.

## Introduction

Osteoarthritis (OA) is a degenerative disease characterized by the progressive deterioration of articular cartilage and structural changes to the entire synovial joint. Also referred to as degenerative joint disease or osteoarthrosis, OA is one of the most prevalent causes of severe pain and chronic physical disability in humans and dogs (Vina and Kwoh, 2018). Since the articular cartilage is hypocellular, avascular and aneural, the turnover of extracellular matrix (ECM) molecules is very slow, which limits its repair capacity (Varela-Eirin *et al.*, 2018). Current palliative treatments, including combinations of non-steroidal anti-inflammatory drugs, analgesics, nutraceuticals, functional foods, physiotherapy and alternative therapies such as acupuncture, often do not provide complete pain relief. Non-pharmaceutical treatment options may also include surgery, weight loss, exercise modification, and physical therapy. Cell therapy and tissue engineering have emerged as potential candidates, using cells such as mesenchymal stromal cells or MSCs (Szychlinska *et al.*, 2017).

MSCs exist in virtually all tissues and play a significant role in tissue repair and regeneration (da Silva Meirelles *et al.*, 2006, 2008). Their high therapeutic potential is mainly due to their capacity to be activated by signals secreted by injured tissues and production of bioactive molecules (da Silva Meirelles *et al.*, 2016). A large number of studies on the biological properties of MSCs have shown their potential for the treatment of OA in humans, by increasing chondrocyte proliferation rates and extracellular matrix production, as well as by their immunomodulatory capacity (Kong *et al.*, 2017). Results from preclinical and clinical studies show that intra-articular injection of MSCs can reduce pain and improve the joint’s function, but also suggest that more studies are required for higher levels of evidence (reviewed in Sasaki *et al.*, 2019; Wang *et al.*, 2019).

The association of MSCs with three-dimensional (3D) scaffolds can increase their therapeutic potential (reviewed in Szychlinska *et al.*, 2019). Many types of biomaterials have been used, in the search for molecules with biological activity. Among them, hyaluronic acid (HA), a large glycosaminoglycan present in the synovial fluid and with reduced concentration in patients with knee OA (Balazs *et al.*, 1967; Altman, 2003), has been investigated. High molecular weight hyaluronic preparation hylan GF-20, a commercially available hydrogel, is a cross-linked preparation of hyaluronan with a molecular weight of 6,000 kDa (Pavelka and Uebelhart, 2011) which has been successfully used as viscosupplementation for knee OA in humans and dogs (Pavelka and Uebelhart, 2011; Shen *et al.*, 2014; Carapeba *et al.*, 2016). However, the effect of viscosupplementation is limited, so that it could be increased and particularly extended for longer periods by the combination with MSCs.

The association of MSCs with HA to treat cartilage has been studied in different species, mostly animal models. Although the treatments are safe and promising results are observed, it is still not possible to draw conclusions on their real benefits, due to the limitations inherent to artificial models and to the heterogeneity and low quality of many of the clinical studies (Roffi *et al.*, 2018). It has been stressed that the use of naturally occurring diseases in companion animals can provide more reliable information in translational medicine (Kol *et al.*, 2015; Hoffman and Dow, 2016). The present work aimed at determining the potential of high molecular weight hyaluronic acid to support the chondrogenic differentiation of canine ASCs *in vitro*, as well as the therapeutic potential of ASCs in combination with HA in dogs suffering from osteoarthritis.

## Materials and Methods

### Animals

Four healthy male dogs submitted to surgical intervention for procedures of castration were used as donors of subcutaneous adipose tissue samples, with prior signing of informed consent by the owners. Twelve dogs of different breeds with bilateral OA of the hip joint were recruited for treatment, as detailed below. The protocol was approved by the Animal Ethics Committee of Universidade Luterana do Brasil (no. 2016.82).

### Reagents, culture media and solutions

Complete culture medium consisted of Dulbecco’s modified Eagle’s Medium (DMEM) supplemented with HEPES (free acid, 2.5–3.7 g/l) and 10% fetal bovine serum (Life Technologies do Brasil, SP, Brazil). Hank’s balanced salt solution containing 10 mM sodium HEPES (HBSS) was used for washing and resuspending the cells. All reagents used in this study were from Sigma-Aldrich Brasil Ltda (SP, Brazil), unless otherwise stated. Plasticware was from Greiner Bio-One Brasil (SP, Brazil).

### Isolation, culture and characterization of canine ASCs

Subcutaneous adipose tissue was digested with collagenase type I, and cultured at 37 °C as previously described (da Silva Meirelles *et al.*, 2006, 2016). Cells between passages 3 and 5 were used for all experiments and for treating OA dogs, as described below. Experiments were done in triplicate.

ASCs were analyzed for morphology, surface phenotype, proliferation and differentiation potential. Cultures were observed under an inverted phase-contrast microscope (Axiovert 25, Zeiss, Hallbergmoos, Germany). Photomicrographs were taken with a digital camera (AxioCam MRc, Zeiss), using the AxioVision 3.1 software (Zeiss). Cell proliferation rate was assessed by counting the number of cells recovered in each passage, as well as the time elapsed. The results are expressed as the number of cells over the days of culture.

The trilineage differentiation potential of ASC cultures was analyzed by incubation for up to 4 weeks with differentiation-inducing culture media (da Silva Meirelles *et al.*, 2016). Adipocytes, osteoblasts, and chondrocytes were revealed with specific staining solutions (Oil Red O, Alizarin Red S, and Alcian Blue, respectively). All procedures had negative control cultures (undifferentiated cultures).

For immunophenotyping, cultures derived from one donor were analyzed in triplicate. The cells were incubated for 30 min at room temperature with antibodies specific for canine CD45, MHC class II or CD90 and conjugated with fluorescein isothiocyanate (FITC) or R-phycoerythrin (PE) (eBioscience, La Jolla, CA, USA). The cells were analyzed on an ACCURI C6 flow cytometer (Becton Dickinson, USA). At least 10,000 events were collected, and the results were analyzed with the BD Accuri C6 software.

### Proliferation and differentiation of ASCs in HA constructs

For encapsulation in high molecular weight hyaluronic acid (Synvisc®, Sanofi Brasil, Brazil), ASCs were resuspended in DMEM at 8 x 10^5^ cells/mL, mixed with HA at 1:10 (v:v) and dispensed into 24-well plates (0.5 ml/well). After 3 h incubation at 37ºC for cell attachment, the scaffolds were transferred to 12-well plates and submerged in control or chondrogenic-inducing medium. The cell/HA constructs were maintained at 37 ºC with 5% CO_2_ for up to 28 days, with medium change every 3 or 4 days.

After 3 days in culture, the proliferation of ASCs encapsulated in HA was determined with a Quant-iT Pico-Green dsDNA kit (Molecular Probes, Invitrogen), which allows quantitating double-stranded DNA. Measurements were performed using a SpectraMax (Molecular Devices, Sunnyvale, CA, USA) plate reader at 485 nm excitation and 535 nm emission wavelengths.

Cultures were photographed as described above on days 7, 21 and 28 for analysis of morphology. On day 28, the constructs were stained with 0.5% Alcian blue (pH 2.5), to detect production of cartilaginous matrix proteoglycans. Samples collected for histological analysis were fixed in 4% paraformaldehyde and embedded in paraffin blocks. Sections of 4-μm thickness were mounted on glass slides and stained with Alcian blue. All sections were evaluated under light microscope. Non-seeded HA scaffolds were also stained, as controls. ASCs from three donors were analyzed, and experiments were done in triplicate.

Sulphated glycosaminoglycans (GAGs) were measured using the dimethyl blue (DMB) technique. ASC-encapsulated constructs were collected on day 28 and digested overnight in phosphate buffer 50 mM pH 6.5 with 0.24 g/L L-cysteine and 0.4% EDTA 0.5 M. After mixing with chloroform and centrifuged at 10,000 x *g* for 15 minutes, 4 °C, 25 μL of the supernatant was mixed with freshly prepared DMB solution (DMB 0.3 mol/L with 2 mol/L Tris) and absorbance was read at 530 nm in a plate reader (SpectraMax M3, Molecular Devices). Protein content was determined using the Lowry method.

### Treatment of dogs with chronic osteoarthritis

To investigate the therapeutic potential of ASCs and ASC/HA constructs in osteoarthritis, ASCs alone or encapsulated in hyaluronic acid were used to treat OA-affected dogs. Twelve adult client-owned dogs were included in the study. Inclusion criteria were age (≤ 6 years old), lameness and pain due to OA associated with bilateral hip dysplasia. Exclusion criteria were previous surgery, any kind of treatment during the previous two months, dislocation of the affected joint and presence of infections, cancer or other diseases which could interfere with interpretation of the results.

The animals were sequentially assigned to three groups (n = 4/group). ASCs and ASC/hyaluronic acid constructs, prepared as described above, were used for all dogs. The control group received a single intra-articular injection of 0.5 mL phosphate buffered saline (PBS) per joint. The ASC group received 5 x 10^6^ cells in 0.5 mL PBS per joint, and the ASC/HA group was treated with 5 x 10^6^ ASCs encapsulated in 0.5 mL hyaluronic acid per joint. Dogs were sedated and injected into single, aseptically prepared sites on both hip joints.

On the first evaluation (day zero), the dogs were examined for clinical, orthopedic and radiological parameters. Clinical and orthopedic results were independently evaluated by two veterinarians, blinded to treatment allocation, on days 7, 30, 60 and 90 after treatment, and another radiographic examination was performed on day 90. The clinical and orthopedic examination used a numerical rating scale, in which lameness, pain on manipulation, articular edema, range of motion and muscle atrophy were scored from 0 to 3 (or to 4, in the case of lameness) according to the severity of symptoms. The detection of crepitus on hip rotation and abduction was also evaluated. The score ranged from 1 (no crepitus) to 5 (crepitus with increasing degree of pain sensation).

Ventrodorsal radiographs were taken on days 0, 30, 60 and 90 and evaluated according to the evidences of instability or degenerative changes of the hip joint. Norberg angle values were measured from the hip extended view (Culp *et al.*, 2006).

### Statistical analyses

Results are expressed as mean ± standard deviation. Data were analyzed and graphs were generated using the Prism 5 software (GraphPad Software Inc, San Diego, CA, USA). Data were tested for normality and analyzed by one-way analysis of variance (ANOVA) followed by Tukey’s post hoc test. A *p*-value less than 0.05 was considered significant for all analyses.

## Results

### Characterization of canine ASCs

**Figure 1:**
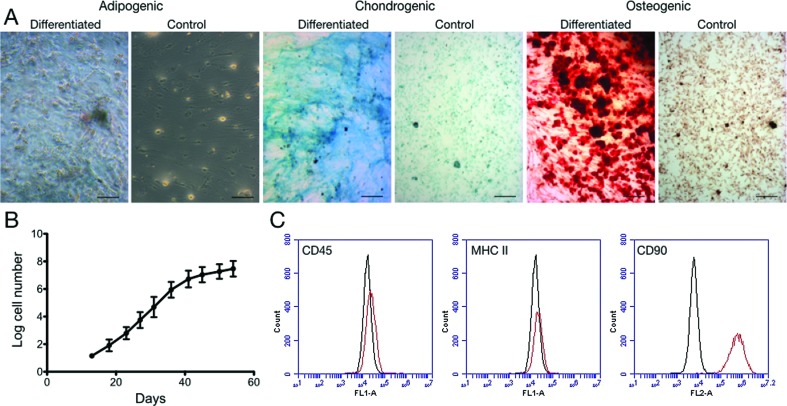
(A) Representative photographs showing the trilineage differentiation capacity of canine ASCs; control cultures were maintained in normal medium. Scale bars, 100 μm. (B) ASC proliferation rate along 60 days of culture. (C) Immunophenotype of canine ASCs (n = 1, analyzed in triplicate). Representative results show that cultures were negative for CD45 and MHC class II, and positive for CD90.

Cells had the characteristic fibroblast morphology of mesenchymal-type stem cells and showed tri-lineage differentiation potential (Figure 1A). The cells expanded rapidly for a period of 40 days, when the expansion rate decreased (Figure 1B). Cells were negative for CD45 and MCH class II antigens, and positive for CD90 (Figure 1C).

### Characterization of ASC/HA constructs

**Figure 2:**
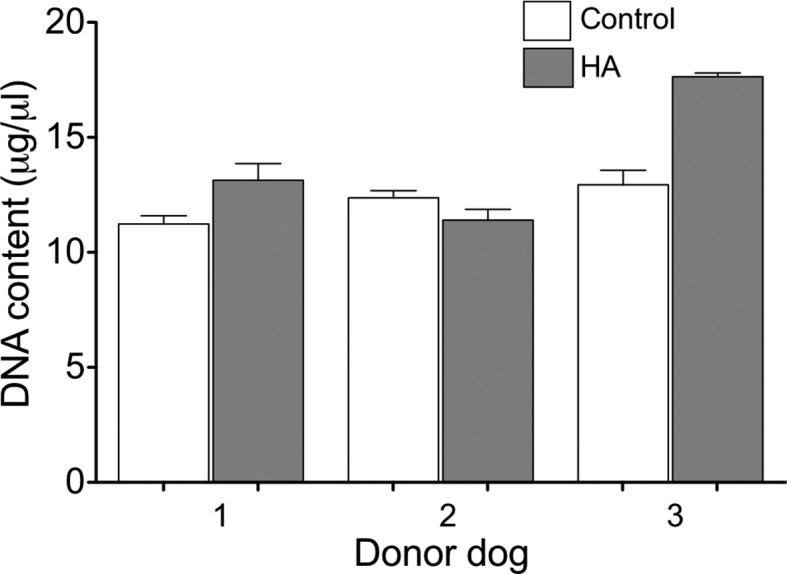
Proliferation rate of canine ASCs cultured for 3 days in normal 2D conditions (Control) or encapsulated in HA, assessed by DNA assay (n = 3).

The proliferation rate of ASCs cultured for 3 days in the HA, as well as control cultures maintained in the conventional 2D system, were fluorometrically assessed by the PicoGreen assay. ASCs from 3 donors were analyzed, and the assays were performed in triplicate. Cells cultured in HA had proliferation rates similar to control cultures (Figure 2).These results were also observed when the GAG contents were determined in ASC-encapsulated constructs cultured for 28 days (Figure 5). GAGs were significantly more abundant in constructs cultured with chondrogenic-inducing medium.[Fig f3] [Fig f4]

**Figure 3 f3:**
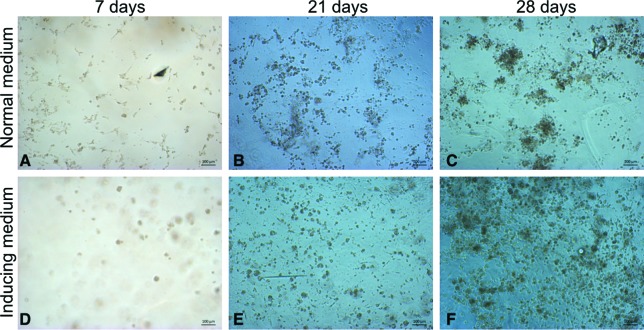
Phase contrast aspect of ASCs encapsulated in HA and cultured for 7, 21 or 28 days in normal medium (A, B, C) or chondrogenic-inducing medium (D, E, F). Scale bars = 100 μm.

**Figure 4 f4:**
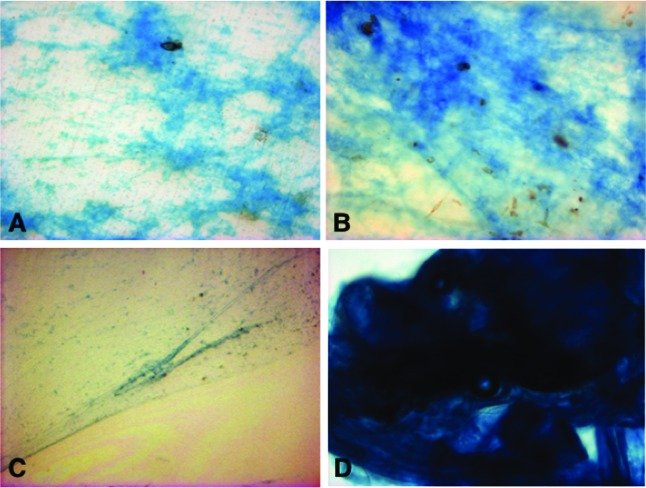
Chondrogenic matrix stained with Alcian blue in encapsulated ASC/HA constructs cultured for 28 days in normal (A) or chondrogenic-inducing (B) medium. A weak background staining can be seen in HA alone (C). (D) Detail of a more dense chondrogenic structure which can also be observed in the constructs. Original magnification x200 (A and B) or x100 (C and D).

**Figure 5:**
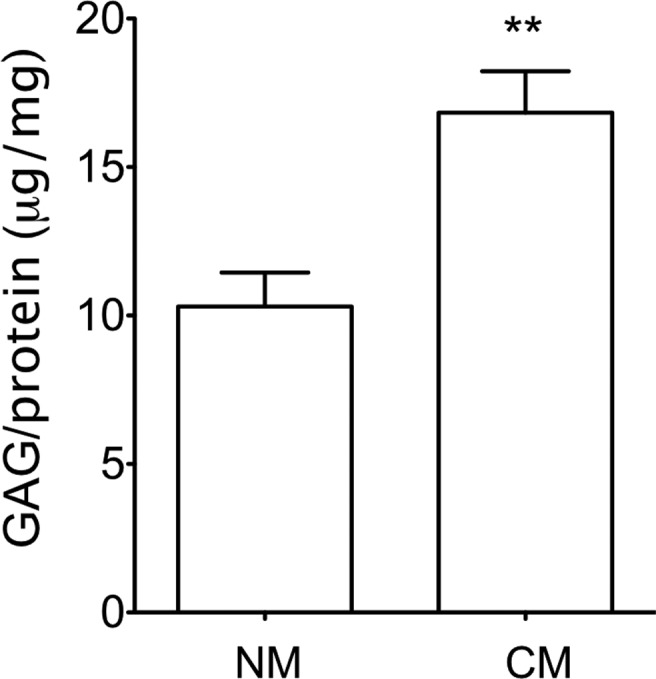
GAG (μg) quantification normalized to protein concentration (mg) of ASC-HA constructs cultured for 28 days (n=3). **p < 0.01.

**Figure 6:**
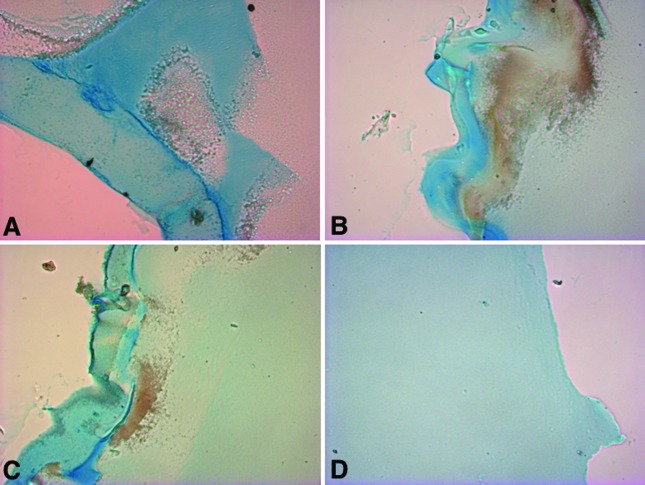
Representative cross-section images of encapsulated ASC/HA constructs cultured for 28 days with chondrogenic-inducing medium, stained with Alcian blue. (A , B, C) Different aspects of the chondrogenic matrix. (D) Control (Synvisc® alone). Original magnification x100.

ASC-encapsulated HA constructs were also analyzed histologically, after 28 days culture in chondrogenic-inducing medium. Sections stained with Alcian blue showed in greater detail the chondrogenic phenotype of the constructs (Figure 6).

### Treatment of dogs with OA

Twelve dogs were included in the study (Table 1). Each joint was considered as an independent sample, considering their individual characteristics, and were treated and assessed individually. The results of the evaluations by two veterinarians on day 0 and days 7, 30, 60 and 90 after treatment, were analyzed as described above.

**Table 1:** Basic characteristics of dogs included in the study.

Group^a^	Breed	Sex	Age	Weight (kg)
Control	German shepherd	Female	5 years	33
	Border collie	Male	3 years	30
	Undefined	Male	11 months	27
	Golden retriever	Female	3 years	30
ASC	Pit bull	Female	5 years	26
	Border collie	Female	7 months	13
	Cimarron	Male	1 year	32
	Rottweiler	Female	1 year	51
ASC/HA	German shepherd	Male	2 years	30
	Undefined	Male	8 months	9
	Golden retriever	Male	2 years	36
	Undefined	Male	3 years	53

a Control: treated with PBS; ASC: treated with adipose tissue-derived mesenchymal stem cells; ASC/HA: treated with ASCs + hyaluronic acid.

**Figure 7:**
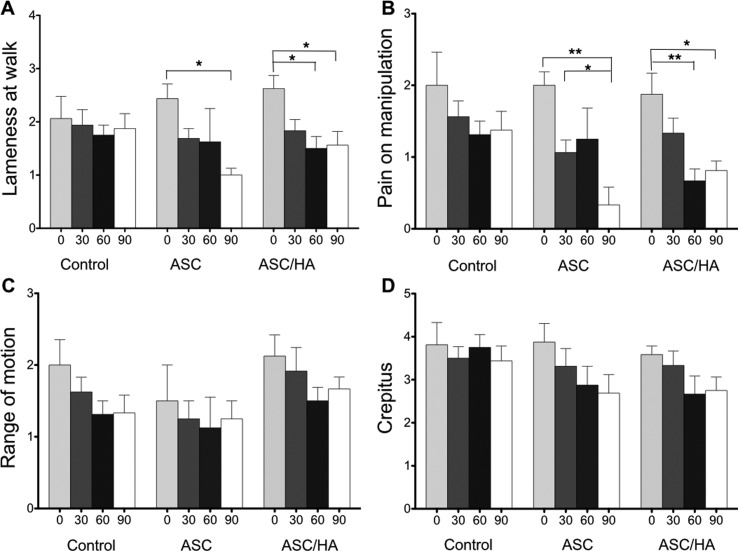
Results of baseline (day 0) or follow-up (days 30, 60 and 90) evaluations of patients in groups control (treated with PBS), ASC (treated with adipose tissue-derived mesenchymal stromal cells) and ASC/HA (treated with ASCs + hyaluronic acid). * p < 0.05; ** p < 0.001, ANOVA and Tukey’s post hoc test.

No significant differences (*p* > 0.05) were observed by investigator for any outcome variable, so the data were pooled together. No adverse events related to the procedure or the treatments were observed during all the follow-up period. Lameness at walk remained unchanged in control animals, but was reduced in the ASC group (*p* < 0.05 on day 90 comparing to day 0) and mainly in the ASC/HA group (*p* < 0.05 on days 60 and 90 comparing to day 0) (Figure 7A). A similar pattern was observed for pain on manipulation, with an earlier (30 days) and more intense effect (*p* < 0.01) in animals treated with ASCs + hyaluronic acid (Figure 7B). Range of motion and detection of crepitus on hip rotation and abduction showed continuous improvement in all groups, but without significant differences (Figure 7C and 7D). For articular edema and muscle atrophy, there were no variations throughout the period in different groups (not shown). Radiographic analyses (not shown), as well as Norberg angle measurements (Table 2), did not show any differences when pretreatment results were compared to results on days 30, 60 and 90.

**Table 2:** Norberg angle measurements before treatment and on follow-up.

Group^a^	Day 0	Day 30	Day 60	Day 90
Control	102.8 ± 9.7	106.0 ± 12.2	106.5 ± 12.1	105.4 ± 14.2
ASC	82.2 ± 14.5	82.1 ± 13.3	83.0 ± 11.3	82.6 ± 12.3
ASC/HA	83.4 ± 10.0	82.5 ± 11.2	83.1 ± 12.2	82.1 ± 11.7

a Control: treated with PBS; ASC: treated with adipose tissue-derived mesenchymal stem cells; ASC/HA: treated with ASCs + hyaluronic acid.

## Discussion

Mesenchymal stem cells have attracted much attention for their potential in tissue engineering, mainly due to their plasticity and secretion of paracrine factors with regenerative functions (Im, 2018). The cells isolated from canine adipose tissue and cultured in the present study were analyzed for morphology, surface profile, proliferation and trilineage differentiation, with results that characterize them as cells of the mesenchymal lineage (Konno *et al.*, 2013). Of particular importance for the treatment of osteoarthritis, canine ASCs differentiated readily into the chondrogenic lineage, similar to previously published results (Marx *et al.*, 2014).

The behaviour of ASCs encapsulated in high molecular weight hyaluronic acid was followed during *in vitro* culture. The cells distributed homogeneously into the HA scaffold, in a 3D configuration. Encapsulation is the more frequently used cell seeding system in this kind of material, since their structure controls cell distribution and ECM diffusion, providing a more adequate environment for tissue engineering (Nicodemus and Bryant, 2008). The 3D cell distribution is important in determining the success of tissue-engineering products, allowing for instance the movement of cells away from the construct to promote chondral integration with the surrounding cartilage (Wu *et al.*, 2018).

Hyaluronic acid has been used to construct hydrogels for cartilage tissue engineering in different combination. A hybrid scaffold combining HA hydrogel with porous poly(e-caprolactone) showed mechanical properties similar to that of human articular cartilage and the ability to maintain the viability, proliferation and phenotype of seeded chondrocytes (Mintz and Cooper, 2014). The proliferation, chondrogenic differentiation and therapeutic potential of mesenchymal stem/stromal cells derived from the bone marrow (de Vries-van Melle *et al.*, 2014) or adipose tissue (Wang *et al.*, 2018) of different species were increased by culture in HA-pNIPAM (poly(N-isopropyl acrylamide) side-chains), HA/Fibrin and HA-PNIPAAm-CL (poly(N- isopropylacrylamide)) (de Vries-van Melle *et al.*, 2014; Wang *et al.*, 2018; Wu *et al.*, 2018), but not in alginate/HA (de Vries-van Melle *et al.*, 2014).

The combination ASC-HA was used for further *in vivo* investigation in dogs with chronic osteoarthritis. Twelve adult dogs of different breeds, with lameness and pain due to OA associated with bilateral hip dysplasia, were treated with a single intra-articular injection of PBS (control group), 5 x 10^6^ allogeneic ASCs (ASC group) or 5 x 10^6^ ASCs encapsulated in hyaluronic acid (ASC/HA group). No modifications in articular edema, muscle atrophy, Norberg angle or radiographic analyses were observed in the 90-day follow-up period. Interestingly, the randomization process allocated dogs with higher NA values to the control group, resulting in a slightly better evolution of this grupo, although the differences were not statistically significant. These results reflect the need for a larger sample number in this kind of study.

Lameness at walk was improved in treated dogs, particularly in the ASC/HA group. A similar pattern was observed for pain on manipulation, with an earlier and more intense effect in ASC/HA animals.

Different animal species have been used in preclinical cell therapy studies, but there are few reports on the use of mesenchymal stem cells to treat spontaneously occurring diseases in veterinary patients. A recent review described only seven reports of cell therapy in dogs, two in cats, and four in horses, published until 2015 (Marx *et al.*, 2015). Among the canine studies, six involved treatment of orthopedic conditions, reporting in general clinical improvement after the therapy (Marx *et al.*, 2015). A more recent study described the treatment of 39 dogs suffering from elbow dysplasia and osteoarthritis with allogeneic ASCs, reporting highly significant clinical improvement after one year (Kriston-Pál *et al.*, 2017). A larger study has also described beneficial effects of ASC treatment in osteoarthritis. Among 203 dogs with degenerative arthritis treated with allogeneic ASCs by intra-articular and/or intravenous injections, 88% showed improvement in clinical symptoms, whereas 12% did not exhibit any change. The response was significantly linked to the age of the animal, with excellent improvement in 90% of young dogs (< 9 years) and 60% of older dogs (Shah *et al.*, 2018). Despite the large sample, this study had no control group and the number of injected cells, or vehicle used, are not described, limiting the significance of the results.

Although positive results have been described with the administration of cells suspended in saline or culture medium, as described above, the use of a carrier such as hydrogels or other types of biomaterials, particularly with chondroinductive properties such as hyaluronic acid, is expected to increase the efficacy of the therapy. Knee viscosupplementation by intra-articular injections of hyaluronic acid is a well established treatment to control primary OA symptoms (Bowman *et al.*, 2018). A few studies have investigated the therapeutic potential of hyaluronic acid in canine osteoarthritis. In a double-blinded trial with 10 dogs with bilateral elbow OA, an intra-articular injection of HA supplemented with methylprednisolone was as effective as autologous conditioned plasma in improving lameness in up to 24 weeks after injection (Franklin and Cook, 2013). The efficacy of the intra-articular hyaluronic acid injection was also compared to traditional conservative treatment in 16 dogs with osteoarthritis. Clinical parameters improved in the HA-treated group from 15 to 90 and 60 to 90 days, compared to the control group (Carapeba *et al.*, 2016).

In humans, some studies have already described the use of HA as a carrier for therapeutic cells in osteoarthritis (Park *et al.*, 2017) and chronic discogenic low back pain (Kumar *et al.*, 2017), or the administration of MSCs followed by hyaluronic acid in osteoarthritic patients (Lee *et al.*, 2012; Lamo-Espinosa *et al.*, 2018), with positive results. To our knowledge, there is only one report of a similar use of ASCs plus HA in dogs with spontaneously developed osteoarthritis. Two dogs with OA of the humeroradial joints received one intra-articular injection of autologous ASCs with Hyalgan (20 mg/2 ml; Fidia), showing functional improvements in lameness and pain on manipulation after one month (Guercio *et al.*, 2012). The combination MSCs/HA has also been tested in dogs with a surgically induced cartilage defect, resulting in more cartilage-like tissue and significant improvement in cartilage lesions than HA alone or saline (Li *et al.*, 2018).

The results of this study suggest a role for ASC/HA constructs in the treatment of cartilage defects, for their chondroinductive properties *in vitro* and effect in the treatment of canine osteoarthritis associated to hip dysplasia. These results contribute also for the development of therapeutic tools for OA in humans. Larger studies with more objective clinical evaluations, such as gait analysis and arthroscopic evaluation, are needed to validate the use of the combination of mesenchymal stromal cells and hyaluronic acid to treat chronic osteoarthritis.
